# Current detection methods of African swine fever virus

**DOI:** 10.3389/fvets.2023.1289676

**Published:** 2023-12-08

**Authors:** Zhiqiang Hu, Xiaogang Tian, Ranran Lai, Xinglong Wang, Xiaowen Li

**Affiliations:** ^1^Shandong Engineering Laboratory of Pig and Poultry Healthy Breeding and Disease Diagnosis Technology, Xiajin New Hope Liuhe Agriculture and Animal Husbandry Co., Ltd., Dezhou, China; ^2^Shandong New Hope Liuhe Co., Ltd., Qingdao, China; ^3^Shandong New Hope Liuhe Agriculture and Animal Husbandry Technology Co., Ltd., (NHLH Academy of Swine Research), Dezhou, China; ^4^China Agriculture Research System-Yangling Comprehensive Test Station, Xianyang, China; ^5^College of Veterinary Medicine, Northwest A&F University, Xianyang, China; ^6^Key Laboratory of Feed and Livestock and Poultry Products Quality and Safety Control, Ministry of Agriculture and Rural Affairs, New Hope Liuhe Co., Ltd., Chengdu, China

**Keywords:** African swine fever virus, detection method, viral genomes, antibodies, laboratory testing, on-site testing

## Abstract

African swine fever (ASF), caused by the African swine fever virus (ASFV), is a highly contagious and notifiable animal disease in domestic pigs and wild boars, as designated by the World Organization for Animal Health (WOAH). The effective diagnosis of ASF holds great importance in promptly controlling its spread due to its increasing prevalence and the continuous emergence of variant strains. This paper offers a comprehensive review of the most common and up-to-date methods established for various genes/proteins associated with ASFV. The discussed methods primarily focus on the detection of viral genomes or particles, as well as the detection of ASFV associated antibodies. It is anticipated that this paper will serve as a reference for choosing appropriate diagnostic methods in diverse application scenarios, while also provide direction for the development of innovative technologies in the future.

## Introduction

African swine fever (ASF), caused by the African swine fever virus (ASFV), is a highly contagious and notifiable animal disease in domestic pigs and wild boars, as designated by the World Organization for Animal Health (WOAH) (1). ASFV is the only member of the *Asfivirus* genus within the *Asfarviridae* family and is the sole arthropod-borne DNA virus (2). The length of the genome exhibits variation among distinct isolates of ASFV, with a range of 170–194 kb (3) and an abundance of over 151 open reading frames (ORFs) (4–6). Notably, strains classified under the ASFV genotype II, such as the seven Polish isolates gathered from 2016 to 2017, demonstrate a high number of ORFs, specifically ranging from 187 to 190 (7). Additionally, the ASFV strain Belgium/Etalle/wb/2018, identified in wild boar in Belgium during 2018, possesses 186 ORFs (8). Based on the sequence alignment analysis of the C-terminal region of the B646L gene, ASFV can be classified into 24 distinct genotypes (9).

The first description of ASFV was done in Kenya in 1921, and then the introduction from Africa to Portugal occurred in 1957, subsequently leading to outbreaks in various European countries (10–12). In 2007, a highly virulent genotype II ASFV emerged in Georgia and rapidly disseminated throughout Eastern Europe and other geographical regions (13). Notably, in China, the detection of genotype II ASFV strains took place in 2018, followed by the identification of genotype I strains in 2021, and the emergence of recombinant strains combining genotypes I and II in 2023 (14–16).

ASFV possesses an intricate icosahedral multilayer architecture, encompassing the envelope, capsid, inner envelope, core shell, and nucleolus (17). It has the capacity to encode 68 structural proteins and more than 100 non-structural proteins (18). Investigating the structural attributes, functionalities, and molecular mechanisms of viral proteins can establish a theoretical foundation for the advancement of diagnostic kits and vaccines. The CD2v protein, which is encoded by the EP402R gene, exhibits characteristics of transmembrane proteins akin to T lymphocyte surface adhesion receptors. It is primarily situated on the external envelope of viral particles and primarily facilitates the binding of the virus to erythrocytes (19, 20). The p72 protein, encoded by the B646L gene, is positioned on the surface of the viral capsid and possesses the ability to induce the host’s production of neutralizing antibodies, as well as actively engage in the viral attachment process to host cells (21). The E183L and CP204L genes encode the p54 and p30 proteins, respectively, which are situated on the inner envelope of viral particles. The functionality of the p54 protein aligns with that of the p72 protein and primarily facilitates virus attachment (22). Conversely, the p30 protein exhibits high expression during the initial phases of infection and plays a crucial role in virus endocytosis (23). Additionally, the CP2475L gene encodes the pp220 protein, which is positioned on the core shell of viral particles and actively facilitates the packaging of the viral core (24). The pA104R and p10 proteins, which are encoded by the A104R and K78R genes, respectively, exhibit localization within the nucleolus of viral particles and play a crucial role in the replication process of the virus (25, 26). Additionally, the multi-gene family MGF (MGF110, MGF300, MGF360, MGF530/505) is associated with host specificity and immune evasion, and possesses distinct functions in determining virulence, facilitating virus replication, and establishing latency (27–29).

The effective diagnosis of ASF holds great importance in promptly controlling its spread due to its increasing prevalence and the continuous emergence of variant strains (30). According to the chapter 3.9.1 of the WOAH Terrestrial Manual (the latest edition: twelfth edition 2023) (31), the laboratory diagnostic procedures for ASF can be categorized into two groups: virus detection and serology. In terms of virus detection, there are several approaches available. Firstly, the isolation of the virus can be achieved by inoculating pig leukocyte or bone marrow cultures. Alternatively, the genomic DNA of the virus can be detected using the polymerase chain reaction (PCR) technique. Another method involves the direct fluorescent antibody test (FAT), where the antigen in smears or cryostat sections of tissues is detected. PCR techniques are particularly valuable for ASFV detection due to their exceptional sensitivity, specificity, rapidity, and applicability in diverse circumstances. Currently the PCR is the most popular technique and can detect ASFV genome from a very early stage of infection in tissues, ethylene diamine tetra-acetic acid (EDTA)-blood and serum samples. In this paper, we mainly discuss the latest research advancements of viral genome or particle detection methods, encompassing laboratory testing techniques (Table 1) such as Real-time fluorescence quantitative PCR (RT-qPCR), Propidium monoazide (PMA)-qPCR, digital polymerase chain reaction (dPCR), as well as on-site testing methods (Table 2) including recombinase polymerase amplification (RPA)/recombinase-aidamplification (RAA), loop-mediated isothermal amplification (LAMP), jumping rolling circle amplification (SRCA), cross-priming amplification (CPA), and clustered regularly interspaced short palindromic repeats (CRISPR). Additionally, as no vaccine is available, the detection of ASFV antibodies serves as a reliable indicator of past infection. Notably, these antibodies are generated within the first week of infection and endure for extended durations, making them a valuable diagnostic tool for identifying the disease, particularly in cases of subacute and chronic manifestations (31). Therefore, the research progress of antibody detection methods (Table 3) is another part of this paper, mainly including enzyme-linked immunosorbent assay (ELISA) and immunochromatographic assay. Above all, we present a comprehensive overview of the existing detection techniques employed for various genes/proteins associated with ASFV, with the objective of offering guidance for the advancement of novel technologies and serving as a point of reference for the selection of suitable diagnostic approaches in diverse application scenarios.

**Table 1:** Detection methods of viral genome by PCR.

Method	Function	Application scenario	Target	Detection limit (evaluation target)	Suitable sample type	Reaction time	References
qPCR	Detection of ASFV genomes	Laboratory testing	MGF505-7R	10 copies/μL (plasmid)	PAM cell, whole blood, nose swab, lymph node tissue	44.33 min	(32)
			E183L	2.63 copies/μL (biological sample)	Whole blood, serum, tissue	27.33 min	(33)
			E248R	10 copies/μL (plasmid)	Serum	24.33 min	(34)
			A137R	10 copies/μL (plasmid)	Whole blood, tissue sample, cloacal swab	27.75 min	(35)
			B646L	1.78 copies/μL (plasmid) 25.2 copies/μL (plasmid)	Tissue	31.17 min	(36, 37)
	Differentiation of genotypes 1 and 2 of ASFV	Laboratory testing	B646L	10 copies/reaction for Genotype I (plasmid) 100 copies/reaction for Genotype II (plasmid)	–	–	(38)
			B646L, E183L	1.07 × 10^2^ copies/μL for B646L (plasmid) 3.13 × 10^4^ copies/μL for E183L (plasmid)	Blood, oral swab, tonsil, and lymph node	–	(39)
			E296R	10 copies/μL (plasmid)	Lymph nodes, spleen, kidney, lung, liver, blood, nasal swab, and environmental swab	23.83 min	(40)
	Differentiation of wild strains and gene-deleted strains of ASFV	Laboratory testing	B646L, I177L, MGF505-2R, EP402R	32.1 copies/μL for B646L (plasmid) 3.21 copies/μL for I177L MGF505-2R and EP402R (plasmid)	Environmental swab, nasopharyngeal swab, blood and tissue	23.50	(41)
			B646L, MGF_360-14 L, CD2v	78.9 copies/μL for B646L 47.0 copies/μL for MGF_360-14 L 82.1 copies/μL for CD2v (plasmid)	Soil, water, blood, fecal sample, table and floor swab, and tissue	45 min	(42)
			MGF-360-12 L, UK, I177L	1.28 copies/μL (plasmid) 2.55 HAD50/mL (biological sample)	Blood	62 min	(43)
			B646L, MGF505-2R	5.8 copies/reaction for B646L (plasmid) 3.0 copies/reaction for MGF505-2R (plasmid)	Serum	22.33 min	(44)
PMA-qPCR	Diagnosis of infectious ASFV	Laboratory testing	B646L	10^1.28^ HAD_50_/mL (virus stock)	Environmental sample	–	(45)
				10^2.32^ HAD_50_/mL (virus stock)	Swine tissue homogenate, swine saliva swab, and environmental swab	3 h	(46)
dPCR	Detecting of ASFV genomes	Laboratory testing	B646L	30.1995copies/reaction (plasmid)	–	109.5 min	(47)
			B646L	4.69 × 10^−1^copies/μL (plasmid)	Tonsil, lymph node, liver, spleen, lung, kidney, and brain	28 min	(48)
			K205R	10 copies/reaction (plasmid)	Excrement sample, serum	80 min	(49)
iiPCR	Detection of ASFV genomes	On-site testing	B646L	20 copies/reaction (plasmid)	Whole blood, serum, heart, lung, spleen, brain, liver, lymph node, tonsil, kidney and nasal swab	25 min	(50)
	Differentiation of genotypes I and II of ASFV		E296R	20 copies/reaction	Lymph node, liver, lung, spleen, serum, and environmental swab	40 min	(51)

**Table 2:** Detection methods of viral genome by multiple-polymerase amplification technologies.

Method	Function	Application scenario	Target gene	Detection limit (evaluation target)	Suitable sample type	Reaction time	References
RPA/RAA	Detection of ASFV genomes	On-site testing	B646L	3.5 copies/μL (the molecular standard ASFV DNA)	Whole blood	–	(52)
				10^2^ copies /reaction (plasmid)	Serum	10 min	(53)
				93.4 copies/reaction for RPA (plasmid) 53.6 copies/reaction for RAA (plasmid)	Blood	16 min	(54)
	Detection of CD2V deletion		EP402R	10 copies/reaction (plasmid)	Oral swab, blood	20 min	(55)
	Detection of ASFV genomes (+LFD)		B646L	200 copies/μL (plasmid)	–	15 min	(56)
				100 copies/reaction (plasmid)	Kitchen waste, swill samples, environment samples from meat stalls and pork product	10 min	(57)
				100 copies/μL (plasmid)	Blood	30 min	(58)
	Detection of ASFV genomes (+nucleic acid test strips)		B646L	10^3^ copies/μL (plasmid)	–	15 min	(59)
	Detection of ASFV genomes (QDMs)		B646L	100 copies/g (plasmid)	Pork	25 min	(60)
LAMP	Detection of ASFV genomes	On-site testing	p10	30 copies/μL (plasmid)	Well-done pork	60 min	(61)
			B646L	10 copies/reaction (plasmid)	EDTA blood, serum, spleen, kidney, liver, tonsil, lymph node and muscle tissue	45 min	(62)
			9GL	13 copies/μL (plasmid)	–	40 min	(63)
			TPII	400 copies/reaction (ASFV-positive serum and blood)	Blood Swab, serum	10 min	(64)
	Detection of ASFV genomes (+CND)		B646L	15.21 copies/μL (unknown)	Tissue, blood, and meat product	–	(65)
	Detection of ASFV genomes (+Hive-Chip)		B646L	30 copies/μL (ASFV synthetic DNAs)	–	70 min	(66)
SRCA	Detection of ASFV genomes	On-site testing	B646L	48.4 copies/μL (ASFV cloned DNA)	Spleen, lymph node, lung, and blood	90 min	(67)
CPA	Detection of ASFV genomes	On-site testing	B646L	7.2 copies/μL (plasmid)	Blood and sera	45 min	(68)
CRISPR	Detection of ASFV genomes (+LAMP)	On-site testing	B646L	7 copies/μL (plasmid)	Nasal swab, spleen, liver, lung, submandibular lymph node and kidney	30 min	(69)
			B646L	1 copies/μL (spiked blood samples)	Whole blood	50 min	(70)
			B646L	2 copies/μL (plasmid)	Serum	-	(71)
	Detection of ASFV genomes (+RPA/RAA)		B646L	20 copies/reaction (plasmid)	Serum	1 h	(72)
			B646L	10 copies/reaction (plasmid)	Blood, Nasopharyngeal swabs, Spleen, Liver, Lung, Kidney	1 h	(73)
	Detection of ASFV genomes (+POC)		B646L	5.7 × 10^4^ copies/μL (synthetic dsDNA)	–	2 h	(74)
Hybridization chain reaction-sensitized magnetic nanoclusters and affinity chromatography	Detection of ASFV genomes	On-site testing	B646L, B962L, C717R, D1133L, and G1340L	1.2 × 10^7^ copies/μL (plasmid)	Serum, swine tissue, feed and lymph nodes	30 min	(75)
Colloidal gold test strip assay	Detection of ASFV particles	On-site testing	P30 protein	2.16 ng (purified recombinant P30 protein)	Serum, plasma, anticoagulated blood, lymph node, spleen, liver	5–7 min	(76)
Chimeric DNA/LNA-based biosensor	Detection of ASFV genomes	On-site testing	B646L	178 copies/μL (plasmid)	Blood	5 min	(77)

**Table 3:** Detection methods of antibodies.

Method	Function	Application scenario	Target viral protein	Sensitivity	References
Indirect ELISA	Distinction of the absence of CD2v, antibody detection in the early stage	Laboratory testing	CD2v, p30	1:5,120	(78)
	Antibody detection in the late stage		pB602L	1: 6,400	(79)
	–		pp62	1:1,600	(80)
	Antibody detection in the early stage		pK205R	1:1,280	(81)
	Antibody detection in the late stage		I329L	1:3,200	(82)
	Antibody detection in the early stage		p11.5	/	(83)
	Distinction of the absence of CD2v		CD2v	1:2,560	(84)
	Antibody detection in the early stage		p22 and p30	1:12,800 1:16	(85, 86)
Blocking (competitive) ELISA	Antibody detection in the late stage	Laboratory testing	p72	1:64	(87)
	Antibody detection in the early stage		p54	1:64	(88)
	Antibody detection in the early stage		p30	1:64–1:512	(89–92)
	Antibody detection in the early stage		pk205R	1:256	(93)
Sandwich ELISA	Antibody detection in the late stage	Laboratory testing	p30, p72	1:1,280 1:12,800	(94, 95)
Colloidal gold test strip	Antibody detection in the late stage	On-site testing	p72	1:64–1:10,000	(96–99)
Microbeads coated with viral proteins	Distinction of the absence of CD2v, Antibody detection in the early stage	On-site testing	CD2v, p30, p54, p22	1:10,240	(100)
Quantum dot-labeled viral proteins	Distinction of the absence of CD2v	On-site testing	CD2v	1: 5.12 × 10^5^	(101)
	Antibody detection in the early stage	On-site testing	p54, p30	1:64,000	(102)
Fluorescent microspheres conjugated with viral proteins	Antibody detection in the early stage	On-site testing	p54	1:1,280	(103)
Luciferase immunoprecipitation system	Antibody detection in the early stage	On-site testing	p30	1:3,200 1:10,240	(104, 105)
	–	On-site testing	p35	1:6,400	(106)
CLIA	Antibody detection in the early stage	On-site testing	p54	1:128	(107)
Nanoplasmonic biosensor technology	Antibody detection in the early stage	On-site testing	p30	1:16,000	(108)

## Viral genome detection by PCR methods

### qPCR

The qPCR method is a frequently employed technique and recommended by WOAH in laboratory diagnostic procedures for ASF. B646L is the target gene recommended by WOAH (109) for ASFV diagnosis and has been wildly used for many years. In the context of further gene structure analysis of ASFV, an increasing number of target genes have been identified for the purpose of ASFV detection. As presented in Table 1, various qPCR methodologies have been devised, utilizing ASFV MGF505-7R gene, E183L gene, 9GL gene, E248R gene, and A137R gene, exhibiting distinct limits of detection (LOD) ranging from 2.63 to 20 copies/μL (32–35). Furthermore, researchers have developed dual qPCR assays targeting the B646L gene, E183L gene, and E296R gene to differentiate between genotype I and genotype II strains of ASFV, in response to the clinical demand for such differentiation (38–40). Furthermore, Chen et al. (36) have successfully developed a triple RT-qPCR detection method, specifically targeting the ASFV p72 gene, which enables differentiation between ASFV, CSFV, and PRRSV. The LOD for ASFV detection using this method is determined to be 1.78 copies/μL. Similarly, Liu et al. (37) have also established a comparable approach for distinguishing the ASFV p72 gene among ASFV, CSFV, and Atypical Porcine Pestivirus, with an LOD of 25.2 copies/μL. The attention towards the CD2v, MGF, and I177L genes has been gradually increasing due to the prevalence of ASFV variant strains and the emergence of gene-deleted vaccines (110, 111). Various qPCR assays, including multiple assays targeting B646L, I177L, MGF505-2R, and EP402R genes (41), triple assays targeting B646L, MGF_360-14 L, CD2v genes (42), triple assays targeting ASFV MGF-360-12 L, United Kingdom, and I177L genes (43), and dual assays targeting B646L and MGF505-2R genes (44), possess the capability to distinguish ASFV wild-type strains from gene-deleted strains.

### Propidium monoazide qPCR

The identification of ASFV infectivity has broad implications in the monitoring of epidemics, evaluating the effectiveness of disinfection measures, and eliminating false negative results (112). To achieve this, researchers have employed the use of ethidium monoazide (EMA) or PMA as a biostain to pre-treat samples. The process and mechanism of EMA/PMA pre-treatment can be described as follows. Initially, the addition of an EMA/PMA solution to samples containing both intact and membrane-compromised cells enables the selective entry of the dye solely into the compromised cells. Subsequently, the dye intercalates into nucleic acids, and the presence of an azide group facilitates a cross-linking between the dye and the DNA upon exposure to intense visible light. Thirdly, the light-induced formation of a highly reactive nitrene radical initiates a reaction with the bound DNA. Fourthly, this alteration significantly impedes the sequential DNA amplification process in polymerase chain reaction (PCR). Ultimately, upon the onset of cross-linking, the light interacts with unbound surplus dye alongside water molecules, leading to the formation of non-reactive hydroxylamine. Therefore, the DNA extracted from cells possessing intact membranes remains unmodified (113). This method has been applied in many microorganisms with membrane structures, including cells, bacteria, fungi, and enveloped viruses. ASFV is an enveloped virus so that some researchers have applied PMA pretreatments to identify infectious viruses from non-infectious ones. As shown in Table 1, Zeng et al. (45) have developed a PMA-qPCR detection method for the rapid diagnosis of infectious ASFV and the evaluation of disinfection efficacy. This method exhibits a detection sensitivity of 10^1.28^ HAD50/mL. With heat evaluation treatments, the detection limit for ASFV is 10^2.28^ HAD50/mL, while with chlorine disinfectants, the detection limit is 10^5.28^ HAD50/mL. Furthermore, Liu et al. (46) has developed a Triton X-100-assisted PMA-qPCR method that allows for the assessment of ASFV infectivity in samples, with a LOD of 10^2.32^ HAD50/mL.

### Digital polymerase chain reaction

dPCR, as originally proposed by Vogelstein B, encompasses droplet dPCR and chip-based dPCR techniques, initially employed for the identification of mutations in the Ras oncogene (114). As presented in Table 1, Jia et al. (47) successfully devised a targeted chip digital PCR (cdPCR) approach for quantifying ASFV by specifically targeting the B646L gene. The LODs for both cdPCR and qPCR approved by WOAH were determined using the same set of primers and the probe with ASFV standard plasmid diluted 10 times as templates, and the results showed that the LOD of the cdPCR method was 30.1995 copies per reaction while the LOD of the qPCR assay was 1,000 copies per reaction, indicating that the cdPCR method is approximately 33-fold more sensitive than the qPCR method endorsed by the WOAH. Wu et al. (49) further advanced the field by developing a droplet digital PCR (ddPCR) method centered on the K205R gene, yielding a minimum detection limit of 10 copies/reaction. Shi et al. (48) conducted a study and engaged in the development of a multiplex dPCR assay to detect ASFV, classical swine fever virus (CSFV), and porcine reproductive and respiratory syndrome virus (PRRSV), achieving a LOD of 4.69 × 10^−1^ copies/μL. In addition to high sensitivity, dPCR can also provide direct absolute quantification. The process of cdPCR is to adopt a sealed chip that partitions samples into thousands of reaction wells to run independent PCR amplifications. Subsequently, the concentration of the target gene in the original sample is calculated by counting and converting positive wells, which have positive amplification of the viral target gene using the Poisson model correction coefficient (115). Therefore, the dPCR method presents itself as a promising instrument for future investigations pertaining to the detection of ASFV mutations.

### Insulated isothermal PCR

The technique of iiPCR utilizing fluorescence hydrolysis probes operates on the principle of Rayleigh-Benard convection and undergoes amplification via cycles of varying temperature gradients (116). As presented in Table 1, Zou et al. (50) have devised an iiPCR approach centered around the B646L gene, exhibiting comparable sensitivity to the WOAH-recommended qPCR and only taking 25 min for on-site testing. Similarly, Song et al. (51) have successfully established an iiPCR assay targeting the E296R gene, enabling swift differentiation of ASFV genotype I and genotype II, with a detection limit of 20 copies/reaction. And the entire process takes only 40 min to complete for on-site testing. Therefore, the iiPCR method is a high-sensitivity and rapid method for ASFV onsite detection and genotype differentiation.

## Viral genome or particle detection by multiple-polymerase amplification technologies

### Recombinase polymerase amplification/recombinase-aidamplification

The concept of RPA technology was initially introduced in 2006 as a means to facilitate the amplification of genome in an isothermal environment, employing recombinases, polymerases, and single-stranded DNA-binding proteins (117). Depending on the specific recombinase utilized, RPA can be further classified as either RPA (T4 phage) or RAA (bacteria/fungi). As presented in Table 2, Ceruti A (52) and Wang et al. (53) have successfully developed a RPA method specifically targeting the B646L gene. This method demonstrates efficient detection capabilities, with completion within a time frame of 10–15 min. The sensitivities achieved are noteworthy, with 3.5 copies/μL and 10^2^ copies/reaction, respectively. Additionally, Fan X (54) has contributed to the field by developing RPA/RAA methods targeting the same B646L gene, exhibiting sensitivities of 93.4 and 53.6 copies/reaction. These methods have shown promising diagnostic consistency when compared to the WOAH real-time fluorescence quantitative PCR. It is important to highlight the work of Wang ZH (55), who has developed an RAA method targeting the EP402R gene. This method allows for the detection of a minimum of 10 copies/reaction and enables the differentiation of CD2V gene deletions. Furthermore, researchers have successfully integrated RPA with various methodologies to enhance the expeditious visual identification of ASFV, thereby augmenting the efficacy of detection. Notably, the amalgamation of RPA with lateral flow chromatographic test strips (LFD) has demonstrated the capability to detect ASFV at a threshold of 1 × 10^2^ copies/reaction (56–58). Similarly, the combination of RPA with nucleic acid test strips (Strip) has proven effective in detecting a minimum of 1 × 10^3^ copies per microliter (μL) (59). Additionally, the integration of RPA with quantum dot microspheres (QDMs) has exhibited the ability to detect ASFV in clinical pork samples, with a sensitivity limit of 100 copies per gram (g) (60).

### Loop-mediated isothermal amplification

The introduction of LAMP technology occurred in the year 2000. This technique entails the design of four primers that specifically target six regions on the desired gene, and employs DNA polymerase for amplification under constant temperature conditions (118). As presented in Table 2, various LAMP detection methods have been developed to target specific viral genes such as K78R, B646L, 9GL, and TPII (61–64), with LODs of 30 copies/μL, 10 copies/reaction, 13 copies/μL, and 400 copies/reaction, respectively. Wang et al. has devised a ladder-shaped melting temperature isothermal amplification (LMTIA) technique that specifically targets the ASFV-B646L gene. This method exhibits the same sensitivity of commercially available qPCR reagent kits with a LOD of 0.5 copies/μL (119). Furthermore, the integration of LAMP with carbon nanodots (CND) technology has facilitated the development of a fluorescent biosensor, enabling the highly sensitive detection of ASFV with a detection sensitivity of 15.21 copies/μL (65). In addition, the LOD for sample analysis using a multiplex and visual detection platform, which combines LAMP with a hive chip (Hive-Chip), is 50 copies/μL (66).

### Clustered regularly interspaced short palindromic repeats

CRISPR/Cas-based nucleic acid detection technology has been developed for ASFV detection. This approach relies on the targeted cleavage of specific sequences by Cas12a/Cas13a proteins, resulting in the release of fluorescence signals from fluorescent reporter probes (120). Integration of CRISPR/Cas technology with RPA or LAMP techniques enables the conduction of the reaction in centrifuge tubes or lateral flow chromatographic test strips, thereby facilitating the visualization of the detection process and subsequent interpretation of the results. As presented in Table 2, multiple methods for ASFV detection have been developed by integrating the CRISPR-Cas12a system with the LAMP method, specifically targeting the B646L gene, with the detection limit ranging from 1 to 7 copies/μL (69–71). Furthermore, the combination of the CRISPR-Cas12a/Cas13a system with RPA/RAA methods demonstrates a detection range of 10–20 copies/μL (72, 73). Notably, He et al. (74) successfully combined CRISPR-Cas12a detection with a fluorescence-based point-of-care (POC) system for ASFV detection, which is based on a CRISPR Cas12a assay to trigger the indiscriminate ssDNA denaturation and a small and sensitive fluorescence-sensing unit with a disposable cartridge to measure the fluorescence signal. Without nucleic acid amplification, the LOD achieved to 5.7 × 10^4^ copies/μL within 2 h. And this compact detection system is automated, integrated, small, lightweight, and inexpensive, ready to be used for on-site ASFV detection or other DNA based pathogens.

### Others

Jumping rolling circle amplification (SRCA) is an emerging isothermal amplification technology that utilizes Bst DNA polymerase (121) and has been employed in the detection methodologies of *Staphylococcus aureus* (122) and *Brucella* (123). As presented in Table 2, the initial development of the SRCA technique for on-site detection was carried out by Milton AAP (67), who employed the ASFV-B646L gene as the target and achieved a LOD of 48.4 copies/μL. This characteristic renders it well-suited for on-site detection of clinical samples.

Cross-priming amplification (CPA) is an isothermal amplification technology that allows for highly specific and sensitive amplification at the constant temperature of 63°C (124). As presented in Table 2, the CPA method was initially devised by Fraczyk et al. (68) for the detection of the ASFV-B646L gene in blood and serum samples, exhibiting a LOD of 7.2 copies/μL.

Lee et al. (75) proposed an optical detection method utilizing the principle of affinity column chromatography to detect ASFV within a time frame of 30 min at ambient temperature, exhibiting a detection limit of approximately 1.2 × 10^7^ copies/μL (Table 2). Zhang et al. (76) devised a sandwich colloidal gold test strip for swift on-site detection of ASFV, employing monoclonal antibodies targeting the P30 protein, with a LOD of 2.16 nanograms (ng) (Table 2). Gomez-Gomez et al. (77) advocated the implementation of a biosensor for the identification of ASFV in porcine blood, demonstrating a LOD of 178 copies/μL (Table 2).

## ASFV associated antibodies detection

### ELISA

ELISA is a detection technique that relies on the specific binding of antigen and antibody. It encompasses various forms such as indirect ELISA, blocking (competitive) ELISA, sandwich ELISA, and multiplex ELISA. As presented in Table 3, in response to the emergence of low virulent ASFV strains with CD2v mutants, Lv et al. (78) developed a dual ELISA approach utilizing CD2v and p30 protein to differentiate wild-type strains from CD2v-deleted strains with low virulence. Furthermore, indirect ELISA methods based on non-structural proteins of ASFV, including pB602L (79), pp62 (80), pK205R (81), I329L (82), p11.5 (83), CD2v (84), p22 and p30 (85, 86), have demonstrated favorable detection capabilities, with an analytical sensitivity ranging from 1:1280 to 1:12800 (Table 3). In comparison to indirect ELISA, the main advantage of blocking (competitive) ELISA is its high sensitivity to compositional differences in complex antigen mixtures, even when the specific detecting antibody is present in relatively small amounts (125). Previous research has demonstrated the successful implementation of blocking (competitive) ELISA techniques based on ASFV structural proteins, including p72 (87), p54 (88), p30 (89–92) and non-structural protein pk205R (93). Wang M (94) and Wang et al. (95) have developed a double-antigen sandwich ELISA utilizing specific enzyme-conjugated antigens, namely p30 protein and p72 protein, exhibiting a sensitivity range of 1:1280 to 1:12,800 (Table 3).

The ELISA method is commonly used to detect antibodies in serum samples. However, the process of collecting serum samples from pigs can induce stress and place a significant burden on personnel. As presented in Table 3, previous research has indicated that antibodies can also be detected in oral fluids, which are easier to collect, suggesting their potential as an alternative sample to serum (126, 127). Mur et al. (128) collected saliva and serum samples from pigs that were inoculated with attenuated ASFV at various time intervals following infection. By utilizing a modified ELISA method, the presence of ASFV antibodies was successfully detected in both saliva and serum samples. Hence, utilizing the recombinant p30 protein, Gimenez-Lirola et al. (129) successfully devised an indirect ELISA method that was capable of effectively identifying antibodies against ASFV in oral fluid samples, and exhibited no significant differences when compared to serum samples.

### Immunochromatographic assay

The immunochromatographic assay is a convenient and rapid technique known for its high specificity and ability to provide visualized results, making it well-suited for on-site detection. As presented in Table 3, several studies have demonstrated that the immunochromatographic assay, utilizing p72 as the labeled protein and colloidal gold as the label, exhibits an analytical sensitivity ranging from 1:64 to 1:10,000 (96–99). Sastre et al. (130) have developed a dual-flow lateral flow assay that is based on the p72 protein of ASFV and the E2 protein of CSFV. This assay specifically detects antibodies against CSFV and ASFV. The implementation of alternative carrier systems for antigen labeling has resulted in the establishment of more efficient methods of detection. For example, the utilization of microbeads coated with recombinant CD2v, p30, p54, and p22 proteins (100), quantum dot-labeled CD2v (101) or p54 and p30 proteins (102), and fluorescent microspheres conjugated with p54 protein (103), can significantly improve the detection rate and stability of positive samples. Additional fluorescent enzyme-linked antibody detection methods have been established in the field of antibody detection. One such method is the luciferase immunoprecipitation system (LIPS) assay, which employs Gaussia luciferase (GLuc) labeled p30 (104, 105) or p35 proteins (106). This assay has been specifically designed for the identification of antibodies against ASFV in pig serum, with the analytical sensitivity ranging from 1:3,200 to 1:10,240. Furthermore, a chemiluminescent immunoassay (CLIA) was developed using the ASFV protein p54 as a serum diagnostic antigen and an anti-p54 monoclonal antibody (107). This CLIA exhibits a sensitivity of 1:128 for the detection of ASFV antibodies. Zhao et al. (108) employed ASFV P30 as the focal point and nanoplasmonic biosensor technology to create an innovative device for detecting ASFV antibodies. This device exhibits a rapid reaction time of 20 min, a remarkable sensitivity of 1:16,000, and the ability to prevent cross-contamination through a simplified one-step sample addition process.

## Discussion and perspectives

Currently, the identification techniques employed for ASFV primarily comprised laboratory testing and on-site rapid testing. Laboratory testing offers notable benefits such as heightened sensitivity, capacity for extensive testing, and cost-effectiveness. However, it necessitates specialized facilities and expensive equipment, rendering it more suitable for large-scale farms rather than routine monitoring in small-scale farms. In contrast, the utilization of on-site rapid testing presents notable benefits, as it allows for direct application in the field, obviating the requirement for specialized facilities or equipment. Nevertheless, it is crucial to acknowledge that the shorter the reaction time required for on-site detection technology, the lower the sensitivity of detection may be. How to improve the sensitivity of detection in a short reaction time may be a promising research direction in the future. Furthermore, it is imperative to recognize that the types of clinical samples significantly impacts the accuracy of detection outcomes. Previous studies have reported that ASFV can also be monitored daily using oral fluids (126), inguinal lymph nodes from dead pigs (131), and ear tissues (132) in addition to blood.

The detection of ASFV antibodies can be utilized as a supplementary approach to evaluate the present infection status of pigs, given the emergence of various ASFV strains (Table 3). Antibody detection targets commonly employed for ASFV infection include structural proteins p72, p54, and p30, as well as non-structural proteins pK205R and pB602L, owing to their diverse functionalities and diverse time of emergence at different stages of viral infection (133). Notably, commercial kits commonly target antibodies against p54 and p30 proteins, which manifest during the initial phases of ASFV infection. Moreover, the viremia exhibited by the attenuated strain of ASFV is characterized by intermittent occurrences (134). Consequently, relying solely on pathogen detection may result in overlooking positive pigs, necessitating the inclusion of antibody detection to overcome this constraint. In subsequent investigations, it may be imperative for researchers to undertake comprehensive examinations of ASFV proteins to devise antibody detection techniques that effectively ascertain the infection stage of pigs, particularly in identifying latent infection carriers.

In forthcoming times, a formidable undertaking persists in the realm of implementing expeditious on-site detection techniques for ASFV in circumstances encompassing import/export quarantine and inter-regional transportation of live pigs. Furthermore, the latent infection attributes of ASF remain incompletely comprehended, and extant detection methodologies lack precision in identifying pigs in a latent infection state. Consequently, the imperative development of methodologies capable of swiftly screening pigs during the latent infection period will be pivotal for the future prevention and control of ASFV.

## Author contributions

ZH: Data curation, Formal analysis, Writing – original draft. XT: Data curation, Formal analysis, Writing – original draft. RL: Data curation, Formal analysis, Writing – original draft. XW: Writing – review & editing. XL: Funding acquisition, Project administration, Validation, Writing – review & editing.
